# Deciphering the interaction between Twist1 and PPARγ during adipocyte differentiation

**DOI:** 10.1038/s41419-023-06283-0

**Published:** 2023-11-23

**Authors:** Leilei Sun, Shaoping Ji, Xuan Xie, Lei Si, Shaohao Liu, Yao Lin, Yahui Wang, Zhenhua Song, Na Fang, Yang An, Jian Yang

**Affiliations:** 1 School of Basic Medical Sciences, Henan University, Kaifeng, 475004 China; 2 Department of Biochemistry and Molecular Biology, Cell Signal Transduction Laboratory, School of Basic Medical Science, Bioinformatics Center, Henan University, Kaifeng, 475004 China; 3 College of Pharmacy and Nutrition, University of Saskatchewan, Saskatoon, SK S7N 5E5 Canada

**Keywords:** Cell biology, Molecular biology, Biochemistry

## Abstract

Obesity, a worldwide epidemic in recent years, is mainly due to the uncontrolled development of adipose tissues, which includes adipocyte hypertrophy and hyperplasia. Adipocyte differentiation is a process involving multiple transcription factor cascades, and the exact mechanism has not yet been defined. As a bHLH transcription factor, Twist1 exerts its activity by forming homo- or heterodimers with other factors. In this study, we showed Twist1 restricts adipogenesis through PPARγ. Expression of various differentiation markers (including PPARγ and adiponectin) and triglyceride-containing lipid droplets were decreased with overexpression of Twist1. Pathway enrichment analysis of RNA-seq data showed that differentially expressed genes (DEGs) caused by Twist1 overexpression were significantly related to lipolysis and PPARγ signaling. This implicates that Twist1 plays important regulatory roles in these processes. ChIP and dual luciferase assays showed that Twist1 could bind either PPARγ or adiponectin promoter to repress their respective transcription or directly to PPARγ protein to regulate its transcriptional activity. Furthermore, Twist1 directly interacted RXRα, which usually forms heterodimer with PPARγ to regulate adipogenesis. Taken together, our results suggest that Twist1 is an inhibitory modulator of adipogenesis and its function is likely through direct interaction with PPARγ protein or its gene promoter.

## Introduction

Obesity and overweight have been emerging to be a health problem worldwide. According to the World Health Organization (WHO), obesity has tripled globally since 1975, and more than 1.9 billion adults and 340 million children were either overweight or obese in 2016 (https://www.who.int/health-topics/obesity). Obesity is a major risk factor for different types of diseases such as diabetes, cardiovascular disease and stroke. Therefore, controlling body weight and reducing obesity would be critical in improving human health and decreasing health care cost.

Adipose tissue is generally divided into white adipose tissue (WAT) and brown adipose tissue (BAT) according to locations and functions. WAT is the predominant type in the body [1]. During the two decades, increasing evidence suggests that adipose tissue is a dynamic organ with metabolic, secretory and thermogenic functions [2]. Abnormal development of adipose tissue may lead to various types of metabolic disorders and/or diseases [3]. Adipogenesis, which is extremely complex and complicated, refers to the process of differentiating preadipocytes into adipocytes. Excess accumulation of differentiated mature adipocytes in the body leads to obesity. Thus, understanding the underlying molecular mechanism of adipocyte differentiation would be highly beneficial in controlling obesity. Adipocyte differentiation, which is influenced by hormones and extracellular environment, is regulated by a cascade of transcription factors [4]. Peroxisome proliferator-activated receptor-γ (PPARγ) and CCAAT/enhancer-binding proteins (C/EBPs) are the key players in the cascade to regulate the activation of adipogenic genes, which, in turn, controls subsequent adipocyte differentiation [5].

PPARγ, which is a ligand-activated transcription factor, exists in three different isoforms (PPARγ_1-3_) due to alternative splicing. It plays a dominant role in adipocyte differentiation [6]. C/EBPβ, EBFs, BMP, Hedgehog and Wnt pathways are upstream of PPARγ [7–9]. Activation of PPARγ facilitates terminal differentiation by inducing the expression of various adipocyte-specific target genes, including those encoding adiponectin, aP2, CD36, LPL and PEPCK [6, 10]. These target genes are essential for triglyceride absorption and storage [6, 10]. Regulation of the target genes by PPARγ is via recruiting and binding co-regulator complexes to the PPAR-responsive elements (PPREs). PPARγ exerts its regulatory role on adipogenesis mainly through forming PPARγ-RXRα heterodimer and binding the heterodimer to PPREs [6].

Twist1 is a transcription factor belonging to the bHLH family. It was first identified in Drosophila as an essential gene in embryonic development [11]. Twist1 is highly conserved through evolution. Twist1 protein consists of three domains, DNA-binding domain, bHLH domain and Twist WR domain [12]. The bHLH domain can form a homodimer or heterodimer with other bHLH factors and contributes to target gene specificity [13, 14]. Twist WR domain regulates transcriptional activity via dimerization. Its dimer recognizes a unique tandem E-box module sequence (CANNTG) in target gene promoter. Twist1 is overexpressed in different types of cancer [15]. Its overexpression enhances epithelial-mesenchymal transition (EMT) and promotes cancer metastasis [12, 16]. Twist1 was also observed to inhibit muscle cell differentiation by dampening myogenic facilitators [17]. Furthermore, Twist1 regulates glycogen storage in skeletal muscle and inhibits adipocyte differentiation [18]. The expression level of Twist1 in WAT is downregulated in obesity but can be restored after weight loss [19]. In BAT, Twist1 can be induced by PPARδ and blocks the transcriptional activity of PPARγ coactivator 1α (PGC-1α) [20]. Ren et al. reported that a regulatory link is likely to be present between Twist1 and PPARγ in mature 3T3-L1 adipocytes [21]. It is clear that Twist1 regulates adipogenesis and likely other functions in adipocytes via PPARγ; however, the underlying mechanism is still unknown. Thus, in the current study, we investigated this regulatory mechanism and showed that Twist1 functioned as a “brake” of adipocyte differentiation. Our current findings may provide a new angle in preventing obesity by harnessing adipogenesis.

## Materials and methods

### Cell culture and differentiation

Mouse fibroblast cell line NIH/3T3, preadipocyte cell line 3T3-L1 and mesenchymal stem cell line C3H10T1/2 were purchased from the American Type Culture Collection (Rockville, MD, USA). For cell lines NIH/3T3 and 3T3-L1, culture media were DMEM (cat. no. 30-2002, ATCC), 10% bovine serum (cat. no. 16170, Gibco, Gaithersburg, MD, USA), and 100 units/ml of penicillin and 100 μg/ml of streptomycin (cat. no. P7630, Solarbio, Beijing, China). For cell line C3H10T1/2, culture media were BME medium (cat. no. 21010, Gibco), 10% heat-inactivated fetal bovine serum (cat. no. 10100, Gibco) and 100 units/ml of penicillin and 100 μg/ml of streptomycin. For each cell line, the cells were cultured in 5% CO_2_ at 37 °C and passaged every 2 days. Upon reaching complete confluence, 3T3-L1 cells were induced to differentiate using the differentiation cocktail (DMEM containing 10% FBS, 0.5 mM IBMX, 5 μg/ml insulin and 1 µM dexamethasone). At day 2, the differentiation medium was DMEM containing 10% FBS and 10 μg/ml insulin. From day 4, the differentiation medium was replaced with DMEM plus 10% FBS and changed every 2 days [22]. For cell line C3H10T1/2, the differentiation cocktail is BME containing 10% FBS, 0.5 mM IBMX (cat. no. I5879, Sigma, Taufkirchen, Germany), 5 μg/ml insulin (cat. no. I9278, Sigma), 1 µM dexamethasone (cat. no. D2915, Sigma) and 1 µM rosiglitazone (cat. no. 71740, Sigma).

### Oil Red O and BODIPY staining

Adipocyte differentiation was monitored by Oil Red O and BODIPY staining. For Oil Red O staining, the steps were: (1) remove cell culture medium and wash cells twice with PBS, (2) add ORO fixative solution to fix the cells for 20–30 min, (3) discard the fixative solution and wash the cells twice with distilled water, (4) soak the cells in 60% isopropanol for 5 min, (5) add ORO stain buffer to the cells and incubate for 10–20 min, (6) discard the dyes and wash the cells 2–5 times with distilled water until no free dye, and (7) observe and photograph the cells under a microscope. For BODIPY staining, the steps were: (1) add 1% paraformaldehyde (PFA) and fix the cells for 30 min, (2) discard the fixative solution and wash the cells 3 times with PBS, (3) dilute BODIPY fluorescent dye probe to PBS at a ratio of 1:1000 and incubate the cells with the diluted dye for 30 min, (4) add Hoechst to stain cell nucleus for 5 min, and (5) observe and photograph the cells under a microscope.

### Vector construction and cell transfection

Promoter of the gene encoding either PPARγ or adiponectin was cloned and ligated into pGL3-Basic luciferase reporter vector. The plasmids were transfected into 3T3-L1 or C3H10T1/2 cells using liposomes. For each cell line, the cells were incubated in opti-MEM to ~70% confluence before being transfected with the plasmid and lipofectamine 3000. Then, culture medium was replaced with fresh medium after 6 h of transfection.

### Lentiviral packaging and transduction

To obtain 3T3-L1 cell line with stable knockdown or overexpression Twist1 (NM_011658.2), lentivirus-mediated Twist1 RNAi or overexpression vector was constructed by Shanghai GenePharma Co. (Shanghai, China) and packaged with corresponding viral capsid. The 3T3-L1 cells were infected with lentivirus carrying Twist1 CDS region or shRNA sequence and screened with puromycin for 2–3 weeks. After screening, the effect on knockdown or overexpression of Twist1 was detected by Western blot.

### RNA extraction and real-time quantitative PCR (RT-qPCR)

Total RNA was extracted from 3T3-L1 cells using Trizol reagent (Takara Bio Inc., Kusatsu, Shiga, Japan) following the manufacturer’s protocol. Purity and concentration of the extracted RNA were detected using a Nanodrop Spectrophotometer (Thermo Fisher Scientific, Waltham, MA, USA). A reverse transcription kit (Vazyme Biotech Co., Nanjing, China) was used to synthesize the cDNA for qPCR. A SYBR Green qRT-PCR kit (Vazyme Biotech Co.) was then used for transcript quantification with specific primers (provided in Supplementary Table S1). The expression level of each target gene was quantified using 2^−ΔΔCt^ method with β-actin as an internal control.

### Cell lysate preparation and Western blot

After washing with precooled PBS for 3 times, the 3T3-L1 or C3H10T1/2 cells were lysed on ice for 30 min using RIPA buffer (Beyotime Biotechnology, Wuhan, China) containing protease inhibitor and phosphatase inhibitor (Bimake, Houston, TX, USA). Upon completion, the cell lysate was centrifuged (12,000 rpm) at 4 °C for 5 min. Protein concentration was measured using a BCA Assay Kit (Beyotime). Samples containing equal amount of proteins were separated by 10% Bis-Tris gels and then transferred to polyvinylidene difluoride membranes (PVDF membranes, Millipore, Amsterdam, Netherlands). After blocking with 5% skim milk in TBST for 1 h at room temperature, the PVDF membranes were incubated with primary antibodies overnight at 4°C and then washed with TBST for 3 times (10 min each time) to remove non-specific antibody bindings. After washing, the membranes were incubated with horseradish peroxidase (HRP)-labeled secondary antibodies for 1 h at room temperature and subsequently washed with TBST. Finally, the antigen-antibody conjugates were detected by the enhanced chemiluminescence (Meilunbio Co., Dalian, China).

### Co-immunoprecipitation (co-IP)

3T3-L1 cells transfected with RXRα-ECFP and PPARγ-EYFP plasmids were washed 3 times with pre-chilled PBS before being lysed on ice for 30 min with NP-40 lysis buffer containing protease and phosphatase inhibitor. Upon complete lysis, the cell lysate was collected and centrifuged (12,000 rpm) at 4 °C for 15 min. The supernatant was subsequently incubated with either anti-GFP mAb conjugated Agarose (avoid heavy and light chain special models, Abmart, Shanghai, China) or anti-Flag-Tag mAb conjugated Agarose (Abmart) at 4 °C overnight. A portion of the sample was taken as an input control. After incubation, the sample was centrifuged at 1000 × *g* under 4 °C for 2 min and the supernatant was discarded. The agarose beads were then washed with pre-chilled NP-40 buffer for 3–5 times to remove any non-specific protein bindings. Finally, the proteins specifically bound to the Agarose beads were eluted with co-IP eluent (Abmart). After sample denaturation and centrifugation, the supernatant was examined on an SDS-PAGE gel.

### Immunofluorescence (IF)

For the immunofluorescence study, 3T3-L1 or C3H10T1/2 cells were first washed with pre-chilled PBS before being fixed with 4% PFA. The cells were then washed and treated with 0.3% Triton X-100 in PBS. After permeation, the cells were washed and the non-specific binding sites were blocked with 2% BSA for 1 h at room temperature. Subsequently, the cells were incubated with the primary antibodies at 4 °C overnight. After washing with 1% BSA, the cells were incubated with the secondary antibodies and Hoechst dye was added to stain cell nuclei for 10 min. Finally, the cells were washed twice with 1% BSA for 5 min before being observed under a fluorescence microscope.

### Chromatin immunoprecipitation (ChIP) assay

ChIP assay is mainly divided into three steps: (1) cell cross-linking and lysis; (2) DNA ultrasonic fragmentation and extraction; and (3) immunoprecipitation. Step 1: For cross-linking, 3T3-L1 cells were treated with 1% formaldehyde at room temperature for 10 min. The cross-linking was terminated by 1.25 M glycine. After discarding the medium and washing twice with cold PBS, the cells were gently scooped up with a cell spatula in the presence of 1 ml cold PBS containing protease inhibitor. The cells were centrifuged to get rid of the PBS buffer and re-suspended in 3 ml FA buffer containing protease inhibitor for lysis (on ice for 15 min). The cell lysate was centrifuged and the supernatant was collected. Step 2: For chromatin fragmentation, cell samples in FA buffer were interrupted by ultrasound. Our pilot experiment showed that length of the DNA cleavage was 200–1000 bp upon ultrasonic interruption for 20 min. Step 3: After preclearing, the DNA samples were incubated with RP-II, Flag antibody or IgG for 2 h at 4 °C, and then protein A/G beads (Santa Cruz, Dallas, TX, USA). The samples were incubated overnight at 4 °C. After centrifugation and washing, LiCl buffer was added to the samples. Samples were further incubated with 300 µl Elution buffer and then centrifuged to collect DNA samples. The genomic DNA was extracted using FastPure Cell/Tissue DNA Isolation Mini Kit (DC102, Vazyme Biotech Co., Nanjing, China).

### Luciferase reporter assay

The promoter of PPARγ or adiponectin was constructed into the firefly luciferase vector pGL3-Basic (Promega, Madison, MI, USA). The empty vector, promoter of PPARγ plasmid or promoter of adiponectin plasmid was co-transfected with pRL-TK plasmid (Promega) into Twist1-OE 3T3-L1 cells by Lipofectamine 3000 reagent (Invitrogen, Waltham, MA, USA). Luciferase activity was measured at 24 h using the TransDetect Double-Luciferase Reporter Assay Kit (TransGen, Beijing, China). Renilla luciferase activity was used as an internal control.

### Statistical analysis

Statistical analysis was performed by GraphPad Prism software and exhibited as means ± SEM. Differences between two groups were assessed by unpaired two-tailed Student’s *t* test. Statistical significance was indicated as follows: *p < 0.05, **p < 0.01 or ***p < 0.001.

## Results

### Expression of Twist1 during adipocyte differentiation

**Fig. 1:**
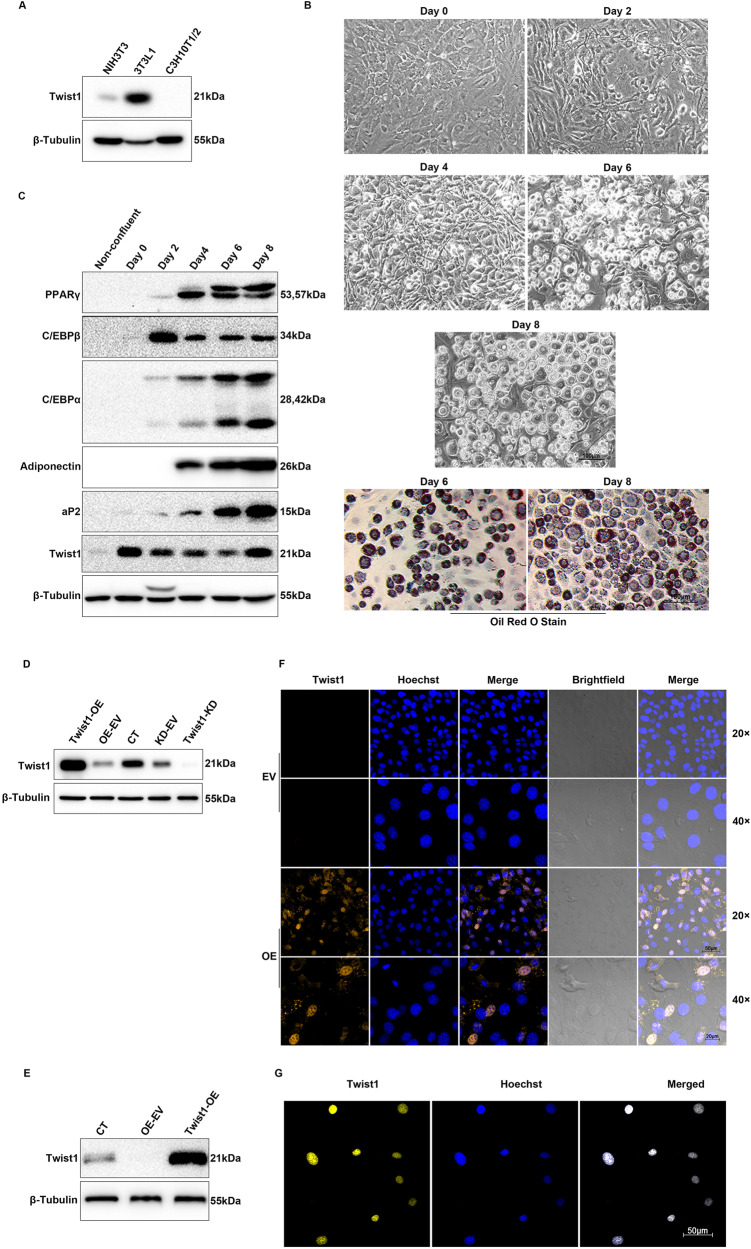
The expression of Twist1 and adipogenic markers during adipocytes differentiation. **A** Detection of Twist1 expression in NIH/3T3, 3T3-L1 and C3H10T1/2 cells by Western blot (WB). **B** Observation of cell morphology at day 0, 2, 4, 6 and 8 after the induced differentiation, and the results of Oil Red O staining at day 6 and 8. **C** Detection of Twist1 expression and adipogenic markers during 3T3-L1 cell differentiation by WB. **D** Overexpression or knockdown of Twist1 in 3T3-L1 cells detected by WB. **E** Overexpression of Twist1 in C3H10T1/2 cells detected by WB. **F** Immunofluorescence assay to detect Twist1 overexpression and localization in 3T3-L1 cells. Magnification ×20 scalebar is 50 µm. Magnification ×40 scalebar is 20 µm. **G** Immunofluorescence assay to detect Twist1 overexpression and localization in C3H10T1/2 cells. Magnification ×20 scalebar represents 50 µm. Twist1-OE Twist1-overexpressing cells, OE-EV OE-empty vector control, CT no treatment control, Twist1-KD Twist1-knockdown cells, KD-EV KD-empty vector control.

Mouse preadipocyte cell line 3T3-L1 has been widely used as a model system to investigate adipocyte development [23]. In this study, we also used 3T3-L1 cells to understand how Twist1 regulates adipocyte differentiation. Adipocyte differentiation is a complex and complicated process that is promoted by a transcriptional cascade. It is sequentially controlled by the expression of a series of adipogenic markers, including PPARγ, C/EBPα, C/EBPβ, adiponectin and aP2 [3]. To elucidate the function of Twist1 in adipogenesis, we determined its expression level, along with the expression levels of the adipogenic markers, during the differentiation of 3T3-L1 preadipocytes. As shown in Fig. 1A, Twist1 was expressed in a much higher level in 3T3-L1 preadipocytes than fibroblast NIH/3T3 cells and C3H10T1/2 stem cells. Upon induction of differentiation, lipid droplets started to accumulate in large quantities over time in the 3T3-L1 cells as evidenced from Oil Red O staining (Fig. 1B). Along progression of the 3T3-L1 cell differentiation, Twist1 expression was downregulated at protein level up to day 6 but rebounded at day 8, while the protein expressions of PPARγ, C/EBPα, adiponectin and aP2 were upregulated (Fig. 1C). Our current result is consistent with previous studies that Twist1 expression was upregulated in mature 3T3-L1 adipocytes [21], implying that Twist1 maintains a regulatory link with PPARγ and/or other adipogenic factors during adipocyte differentiation.

### Negative modulation of adipocyte differentiation by Twist1

To identify the role of Twist1 in adipogenesis, overexpression and interference (knockdown) vectors of Twist1 were constructed and packaged into lentivirus particles. After infection and puromycin selection, cellular proteins were extracted to detect the expression of Twist1 in 3T3-L1 and C3H10T1/2 cells. Compared to control, the expression of Twist1 was upregulated in Twist1-overexpressing cells (Twist1-OE) and downregulated in Twist1-knockdown cells (Twist1-KD) (Fig. 1D, E). Moreover, immunofluorescence (IF) staining showed that Twist1 was primarily localized inside the nuclei (Fig. 1F, G).

**Fig. 2:**
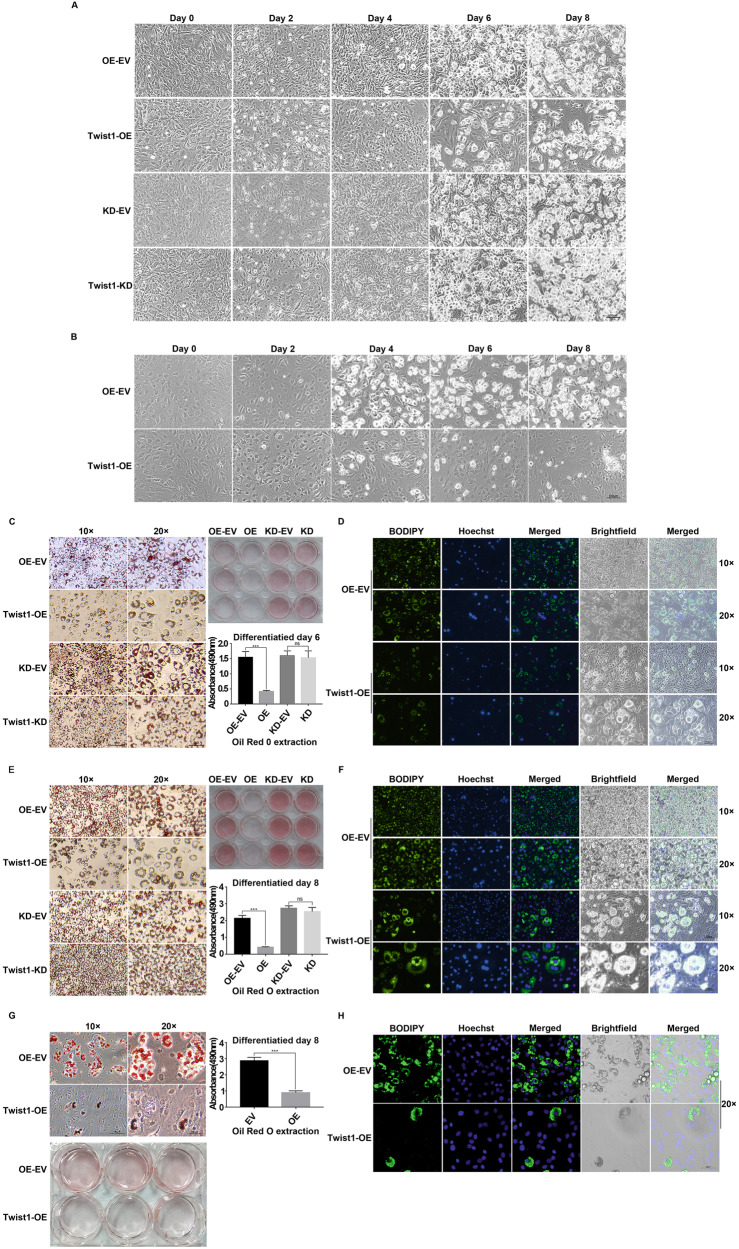
Morphological observation and triglyceride-specific staining revealed that Twist1 inhibits adipocyte differentiation. **A** Morphological observation at day 0, 2, 4, 6 and 8 of Twist1-OE or Twist1-KD cells after induced 3T3-L1 differentiation. **B** Morphological observation at day 0, 2, 4, 6 and 8 of Twist1-OE cells after induced C3H10T1/2 differentiation. **C**, **E** Oil Red O staining of 3T3-L1 cells at differentiation day 6 and 8 was observed under microscope, respectively, and the triglyceride content was statistically analyzed through Oil Red O extraction, the absorbance of which was measured at 490 nm. **D**, **F** BODIPY staining of 3T3-L1 cells at differentiation day 6 and 8, respectively. **G** Oil Red O staining of C3H10T1/2 cells at differentiation day 8 was observed under microscope, and the triglyceride content was statistically analyzed through Oil Red O extraction, the absorbance of which was measured at 490 nm. **H** BODIPY staining of C3H10T1/2 cells at differentiation day 8.

**Fig. 3:**
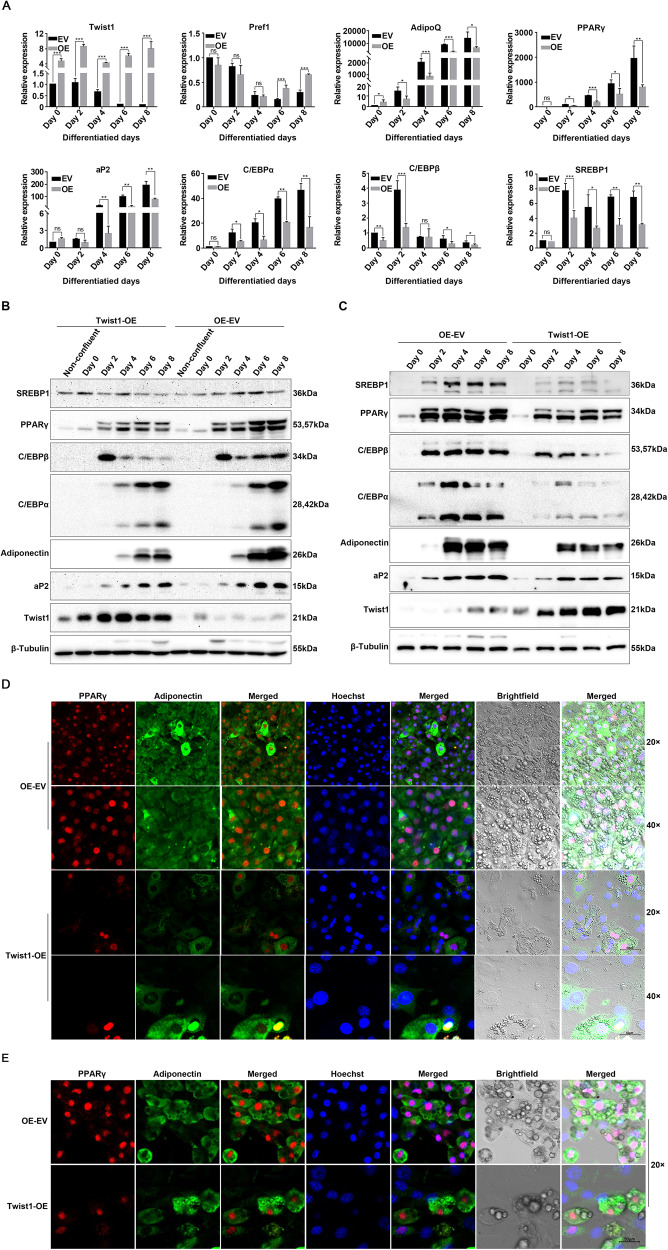
Q-PCR and WB verifications of the inhibitory effect of Twist1 on adipocyte differentiation. **A** Detection of the mRNA levels of adipogenic markers in the differentiation process of Twist1-OE 3T3-L1 cells by Q-PCR. **B**, **C** Detection of the protein levels of adipogenic markers during the differentiation of Twist1-OE 3T3-L1 and C3H10T1/2 cells by WB, respectively. **D**, **E** Immunofluorescence assay to detect expression and localization of PPARγ and adiponectin in Twist1-OE 3T3-L1 and Twist1-OE C3H10T1/2 cells, respectively. The red fluorescence-labeled and green fluorescence-labeled secondary antibodies were used to detect PPARγ and adiponectin, respectively. The nucleus was stained with Hoechst. Magnification ×20 scalebar represents 50 µm, and ×40 scalebar represents 20 µm.

We further investigated the differentiation of Twist1-OE and Twist1-KD 3T3-L1 preadipocyte cells (Fig. 2). As shown in Fig. 2A, C–F, the content of lipid droplets was much lower in Twist1-OE 3T3-L1 cells than that of 3T3-L1 cells with empty vector control since day 4. No significant difference was observed between the empty vector control and the Twist1-KD 3T3-L1 cells. At day 8, majority of the Twist1-KD or control cells had completely differentiated into mature adipocytes full of large lipid droplets, while the Twist1-OE cells exhibited incomplete differentiation as evidenced by Oil Red O and BODIPY staining. For the C3H10T1/2 cells, we observed similar results that Twist1-OE cells were barely differentiated at day 8 (Fig. 2B, G, H). The level of triglycerides was evaluated in the adipocytes via Oil Red O extraction and quantification. It is clear the content of triglycerides was significantly lower in Twist1-OE 3T3-L1 and Twist1-OE C3H10T1/2 cells than the empty vector controls (Fig. 2C, E, G), implicating that Twist1 suppressed adipocyte differentiation. We further measured the expression levels of adipogenic transcription factors by qPCR and Western Blot (Fig. 3). Compared to the empty vector control, the expressions of adiponectin (AdipoQ), PPARγ, aP2, C/EBPα, C/EBPβ and SREBP1 were significantly decreased at mRNA level in Twist1-OE 3T3-L1 cells; however, the mRNA level of Pref1 was increased (Fig. 3A). Downregulations of PPARγ, C/EBPα, C/EBPβ, aP2 and adiponectin were further confirmed at protein level by Western Blot in Twist1-OE 3T3-L1 and Twist1-OE C3H10T1/2 cells (Fig. 3B, C). Protein level of SREBP1 was significant downregulated in Twist1-OE C3H10T1/2 cells, however, no significant alternation in protein level of SREBP1 was observed in Twist1-OE 3T3-L1 cells. To further confirm our observations, we also assessed the protein level and cellular localization of PPARγ and adiponectin by immunofluorescence staining (Fig. 3D, E). Consistently, the protein levels of nuclear PPARγ and cytoplasmic adiponectin were much lower in Twist1-OE 3T3-L1 and Twist1-OE C3H10T1/2 cells. Therefore, we concluded that Twist1 is a negative modulator of adipogenesis and overexpression of Twist1 suppresses adipocyte differentiation.

### Differentially expressed genes (DEGs) and pathway enrichment analysis of differentiated Twist1-OE adipocytes

**Fig. 4:**
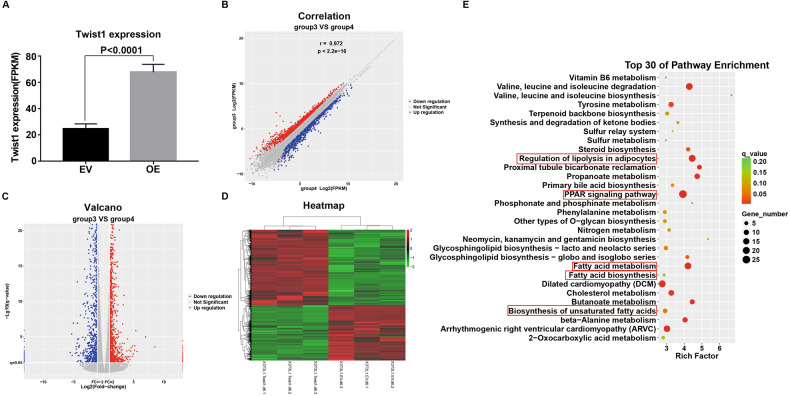
Pathway enrichment analysis of Twist1-regulated differentially expressed genes (DEGs) during adipocyte differentiation by RNA-seq. **A** Twist1 overexpression revealed by RNA-seq. **B**, **C** Correlation analysis and volcano plot of DEGs. **D** Heatmap of DEGs. **E** Pathway enrichment analysis of DEGs.

**Fig. 5:**
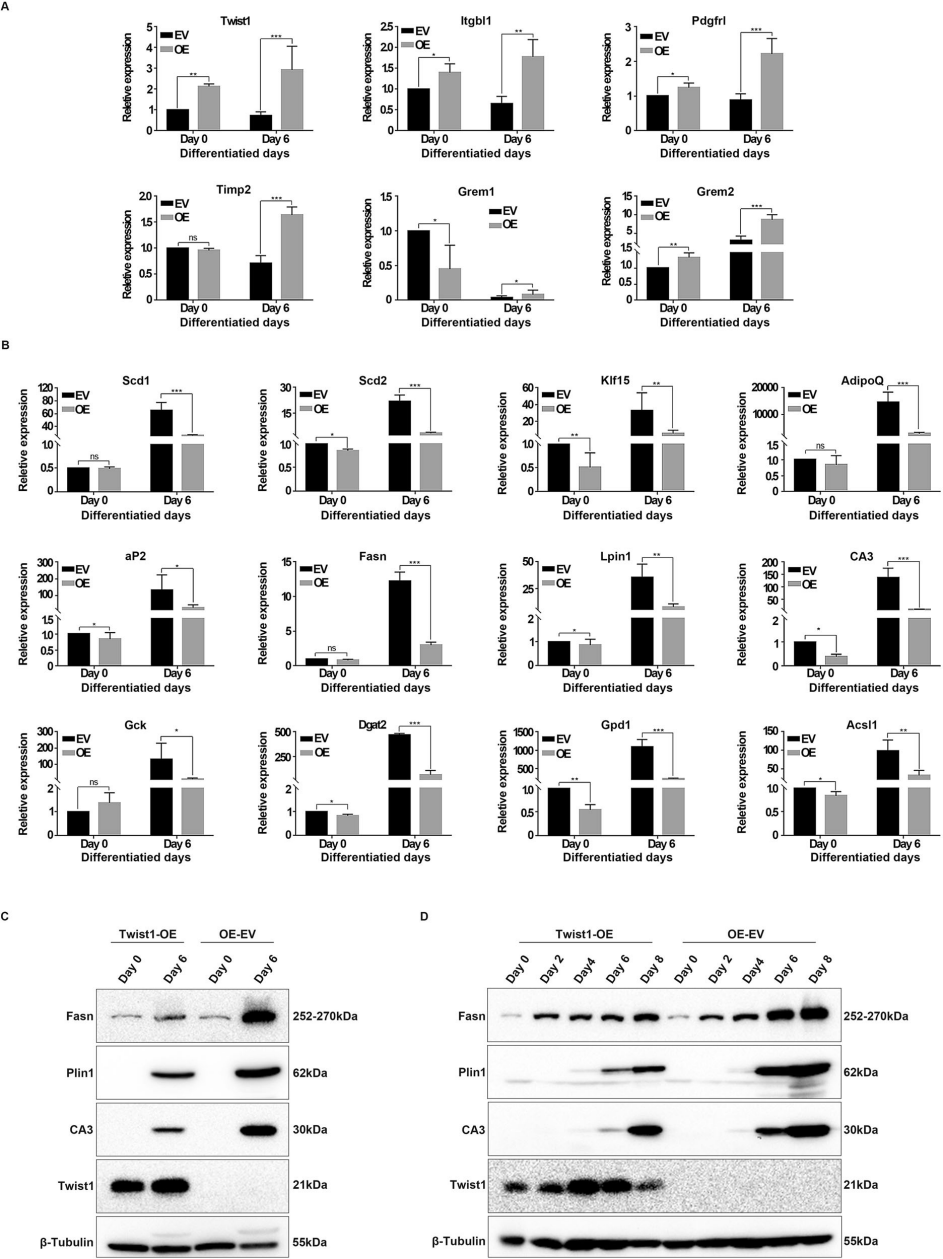
Verification of DEGs regulated by Twist1. **A**, **B** Detection of the mRNA levels of DEGs up-regulated and down-regulated by Twist1 using Q-PCR. **C**, **D** Detection of the protein levels of DEGs down-regulated by Twist1 during adipocyte differentiation using WB.

To elucidate the underlying molecular mechanism of the inhibitory effect of Twist1 on adipogenesis, we evaluated gene expression profiling and key regulatory pathways in the differentiated Twist1-OE 3T3-L1 cells (day 6) by RNA-seq (Fig. 4). Pathway enrichment analysis of the expression profiling data showed that Twist1 was significantly related to lipolysis regulation, PPARγ signaling pathway, fatty acid metabolism and biosynthesis in adipocytes (Fig. 4E), indicating that Twist1 is likely to play critical roles in these processes. To further verify the RNA-seq results, we measured the mRNA levels of differentially expressed genes (DEGs) regulated by Twist1 using Quantitative real-time PCR (Q-PCR). The measured DEGs included up-regulated genes Itgbl1, Pdgfrl, Timp2, Grem1 and Grem2, and the down-regulated genes Scd1, Scd2, Klf15, AdipoQ, aP2, Fasn, Lpin1, CA3, Gck, Dgat2, Gpd1 and Acsl1. Our Q-PCR results were consistent with RNA-seq (Fig. 5A, B). Some of these DEGs have been previously investigated. Itgbl1 and Pdgfrl are proposed to be preadipocyte markers [24]; expression of Timp2 is decreased during adipocyte differentiation [25]; Gremlin1 and 2 (Grem1 and Grem2) are negative adipogenic markers [26]; stearoyl-CoA desaturase 1 and 2 (Scd1 and Scd2) function as differentiation markers [24, 27]; and Klf15 plays an essential role in adipogenesis through its regulation of PPARγ [28]. Our current RNA-seq and Q-PCR results showed that negative adipogenic factors were up-regulated by Twist1 (Fig. 5A) and pro-adipogenic factors was down-regulated by Twist1 (Fig. 5B), which confirmed the inhibitory effects of Twist1 on adipocyte differentiation. Furthermore, we verified the protein expression levels of three down-regulated genes (Fasn, Plin1 and CA3) involved in lipid metabolism and adipose development in the differentiated Twist1-OE cells (Fig. 5C, D). Consistently, the protein expressions of these three genes were decreased during adipocyte differentiation in Twist1-OE 3T3-L1 cells. Finally, we did pathway enrichment analysis of the DEGs responsive to Twist1 and identified that PPARγ signaling was significantly enriched. This implicates that a link is present between Twist1 and PPARγ signaling.

### Twist1 regulates adipocyte differentiation via inhibiting PPARγ signaling

**Fig. 6:**
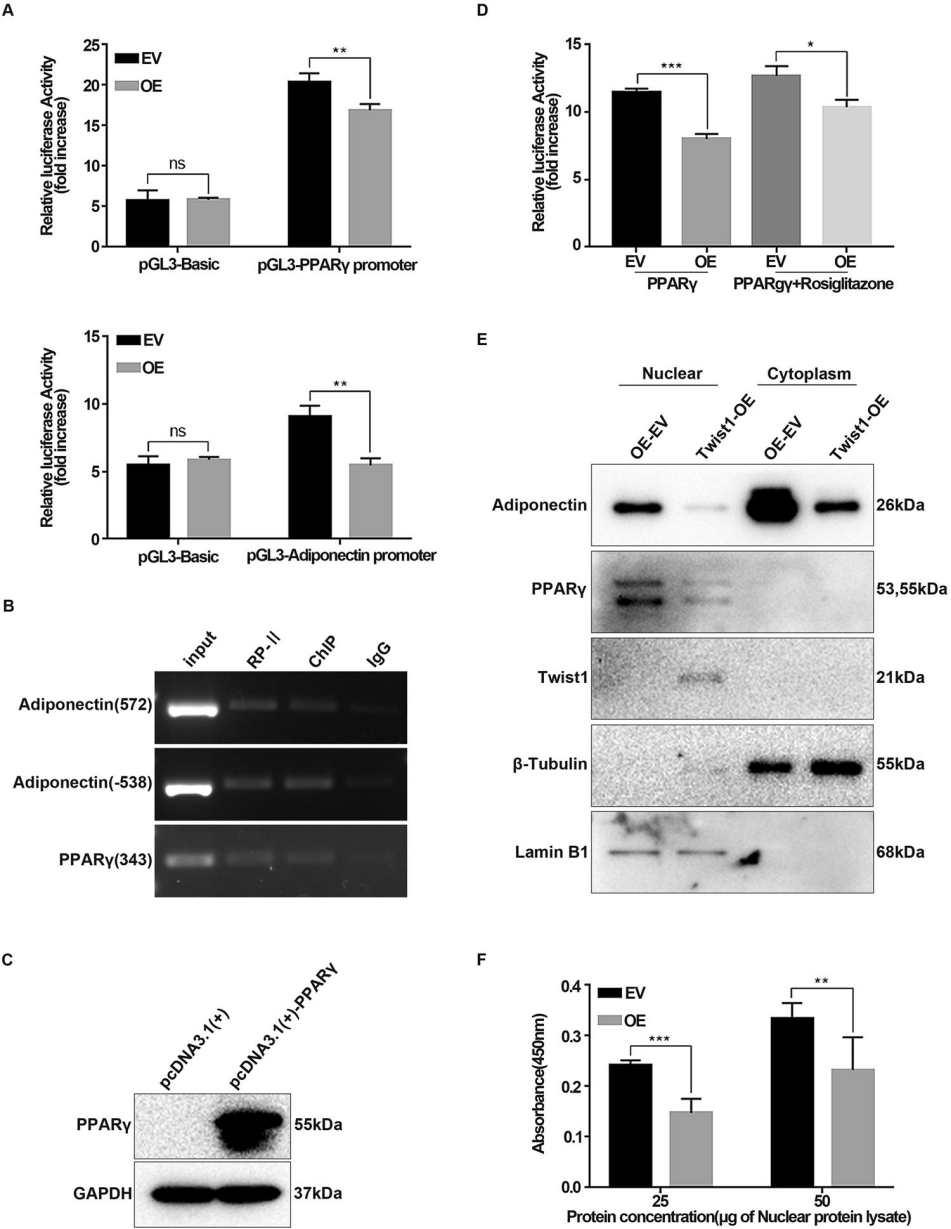
Twist1 regulates adipocyte differentiation via inhibiting PPARγ signaling. **A** Transcriptional regulation of PPARγ or adiponectin by Twist1 was detected using dual luciferase reporter assay. pGL3-Basic: the empty vector control. **B** ChIP assay detected the binding sites of Twist1 to PPARγ or Adiponectin promoter. PR-II: RNA polymerase, positive control and IgG: negative control. **C** Overexpression of PPARγ was assessed by WB in Twist1-OE 3T3-L1 cells after being transfected with pcDNA3.1(+)-PPARγ plasmids. pcDNA3.1(+): empty vector control. **D** Effect of Twist1 overexpression on the transcriptional activity of PPARγ was examined with or without rosiglitazone by dual luciferase reporter assay. **E** Nuclear and cytoplasmic extraction of Twist1-OE 3T3-L1 cells at differentiation day 8. Lamin B1: nuclear marker and β-Tubulin: cytoplasm marker. **F** PPARγ transcriptional activity was measured in Twist1-OE 3T3-L1 cells at differentiation day 8 by ELISA after nuclear-cytoplasmic separation. The *x*-axis represents the concentration of the extracted nuclear protein in µg.

The aforementioned studies points to a close link between Twist 1 and PPARγ during adipocyte differentiation. To decipher this link, we decided to evaluate the transcriptional regulations of PPARγ and adiponectin by Twist1 using the dual luciferase reporter gene assay (Fig. 6). Compared to controls, the luciferase activity driven by PPARγ or adiponectin promoter decreased in Twist1-OE 3T3-L1 cells, indicating that Twist1 suppressed the transcriptions of PPARγ and adiponectin (Fig. 6A). Then, we investigated whether Twist1 could bind to the promoter of PPARγ or adiponectin by chromatin immunoprecipitation (ChIP). As shown in Fig. 6B, Twist1 bound to the promoters of PPARγ and adiponectin at the predicted binding site (PPARγ: 343 and adiponectin: 538 and 572). Furthermore, we constructed the PPARγ expression vector (Fig. 6C) and aP2 promoter vector in order to analyze the transcriptional activity of PPARγ. The aP2 promoter vector contains PPARγ responsive element (PPRE) in front of the luciferase CDS region, and thus its luciferase activity is responsive toward PPARγ binding. It was clear that the luciferase activity driven by the aP2 promoter was significantly decreased in Twist1-OE 3T3-L1 cells upon PPARγ binding (Fig. 6D), implicating that the transcriptional activity of PPARγ to aP2 was repressed by Twist1. Since rosiglitazone is a PPARγ agonist [29], we evaluated whether it could rescue the suppression of PPARγ transcription by Twist1 (Fig. 6D). Upon rosiglitazone stimulation, the reduced luciferase activity driven by aP2 promoter was partially rescued in the present of PPARγ. Thus, we may conclude that Twist1 suppresses adipocyte differentiation through inhibiting the expressions of PPARγ and adiponectin and abating the transcriptional activity of PPARγ.

To examine the inhibitory effect of Twist1 on PPARγ transcriptional activation capacity, we performed the nuclear and cytoplasmic extraction and detected the transcriptional activity of PPARγ derived from nuclear component using the PPARγ transcription factor assay kit, which measures DNA-binding activity based on ELISA with PPRE coated on the plate. β-Tubulin and Lamin B1 were used as the cytoplasmic and nuclear makers, respectively, to verify the extraction quality. As shown in Fig. 6E, Twist1 and PPARγ were present in the nuclear component while adiponectin was present in both nuclear and cytoplasmic components. This observation is consistent with the subcellular localization results (Fig. 3D). Protein lysate derived from the nuclear extract was incubated on the assay plate. Intensity of PPARγ binding to PPRE was measured by antigen-antibody reaction and subsequent chemiluminescence. Compared to the empty vector control, the DNA-binding activity of PPARγ to PPRE was significantly decreased in the differentiated Twist1-OE 3T3-L1 cells at day 6 (Fig. 6F), indicating that the transcriptional activation capacity of PPARγ is inhibited by Twist1. In summary, Twist1 can bind to the promoters of PPARγ and adiponectin and significantly decrease the transcriptional activity of PPARγ.

**Fig. 7:**
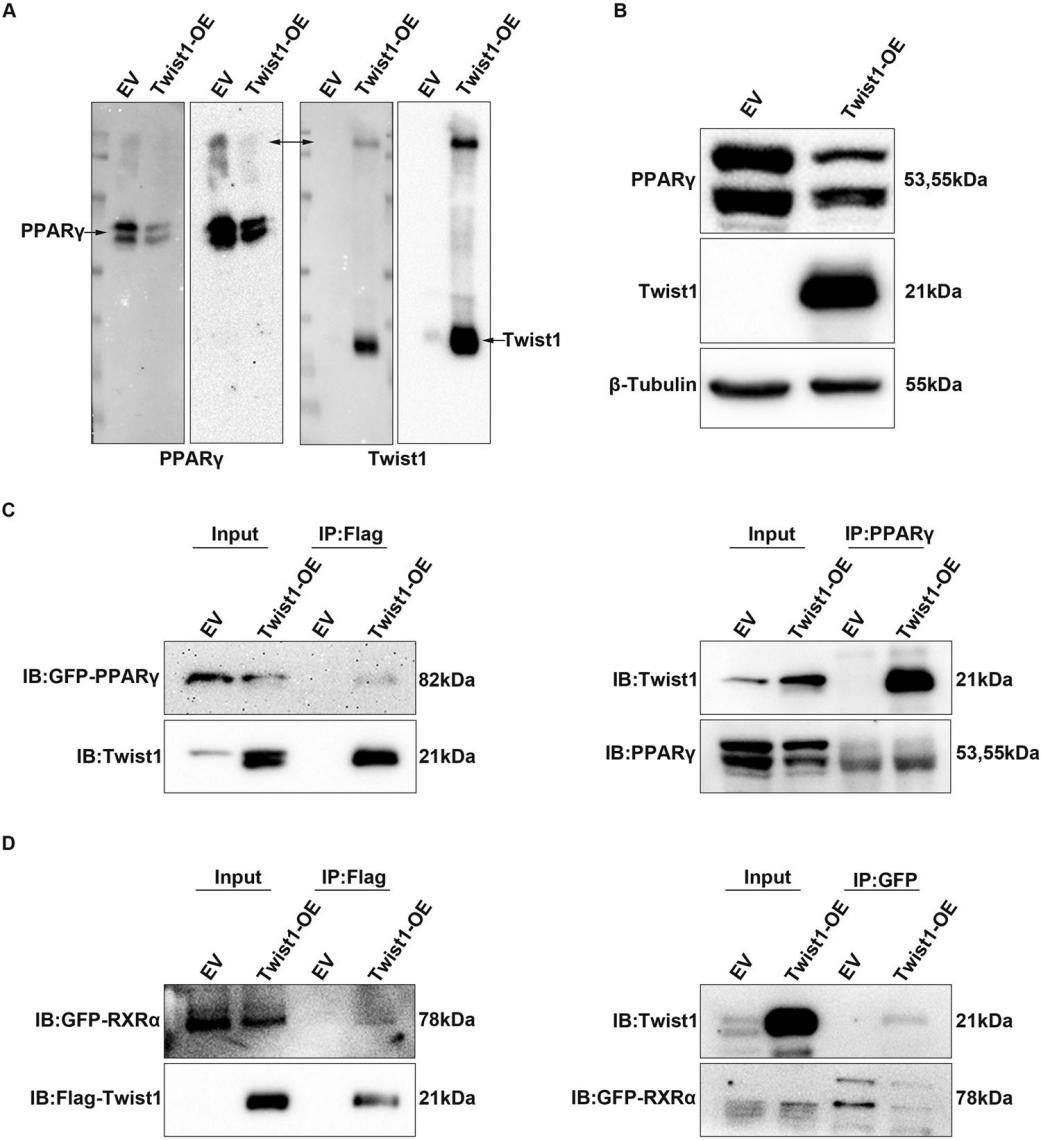
Twist1 directly interacts with both PPARγ and RXRα in 3T3-L1 cells. **A** Non-denaturing electrophoresis to detect the binding between Twist1 and PPARγ. **B** Expressions of Twist1 and PPARγ detected by WB at day 8 of differentiation. **C** Direct interaction between Twist1 and PPARγ assessed by co-IP assay. Twist1-OE 3T3-L1 cells were transfected with pcDNA3.1(+)-PPARγ-ECFP plasmids. Cells were subsequently lysed and immunoprecipitated (IP) with anti-Flag antibody and immunoblotted (IB) with anti-GFP or anti-Twist1 antibody or IP with anti-PPARγ antibody and IB with anti-Twist1 or anti-PPARγ antibody. **D** Direct interaction between Twist1 and RXRα examined by co-IP assay. Twist1-OE 3T3-L1 cells were transfected with pcDNA3.1(+)-RXRα-EYFP plasmids. Cells were subsequently lysed and IP with anti-Flag antibody and IB with anti-GFP or anti-Flag antibody or IP with anti-GFP antibody and IB with anti-Twist1 or anti-GFP antibody.

To investigate direct protein-protein interaction (PPI) between Twist1 and PPARγ, we performed non-denaturing electrophoresis and co-immunoprecipitation (co-IP) assay. Total proteins were extracted from Twist1-OE 3T3-L1 and control cells at day 8 of differentiation. Non-denaturing electrophoresis was applied to detect potential interaction between Twist1 and PPARγ. Twist1 and PPARγ bands appeared at the same location, suggesting that they might bind each other (Fig. 7A, B). Then, Co-IP assay was used to confirm the interaction between Twist1 and PPARγ. Twist1-OE 3T3-L1 and control cells were transfected with PPARγ-EYFP plasmids and cell lysates were co-immunoprecipitated by anti-GFP mAb (agarose conjugated) or anti-Flag-Tag antibody followed SDS-PAGE. Since Twist1 was coupled with Flag-Tag in Twist1-OE lentivirus, Flag antibodies were used to immunoprecipitate Twist1, and GFP antibodies were used to immunoprecipitate PPARγ. The anti-GFP and anti-Twist1 antibodies detected PPARγ and Twist1 in the complex co-precipitated by anti-Flag or anti-GFP Ab, respectively, implicating that Twist1 directly interacted with PPARγ (Fig. 7C). Since PPARγ has been reported to form heterodimers with retinoid X receptors (RXRα) and activate adipocyte-specific aP2 enhancer [30], we evaluated whether Twist1 could also directly interact with RXRα. Twist1-OE 3T3-L1 and control cells were transfected with RXRα-ECFP plasmid and cell lysates were co-immunoprecipitated by anti-GFP mAb (agarose conjugated) or anti-Flag-Tag antibody and followed by SDS-PAGE. Flag and GFP antibody were used to immunoprecipitate Twist1 and RXRα in the cell lysates, respectively. Anti-GFP and anti-Twist1 antibodies detected RXRα and Twist1 in the complex co-precipitated by anti-Flag or anti-GFP Ab (Fig. 7D). Thus, Twist1 can also directly interact with RXRα.

## Discussion

The increasing prevalence of obesity and related diseases has emerged to be a research focus [31, 32]. Obesity is characterized by expanded adipose tissues and metabolic disorders. It becomes a major risk factor of various diseases even cancers [3, 33–35]. Adipose tissue is mainly composed of adipocytes, which are full of triglycerides droplets. Adipose tissue expansion is mediated by hypertrophy (increase in adipocyte size filled with more triglycerides droplets) or hyperplasia (increase in adipocyte number), but excessive uncontrolled adipose development becomes a risk factor for both human health condition and quality of life [3, 31, 36]. Harnessing adipogenesis may be an effective strategy to prevent obesity. Adipocyte differentiation is controlled by a regulatory cascade of transcription factors [37, 38], with PPARγ, a vital adipogenic regulator, located at the core of the transcriptional cascade [37, 39]. PPARγ is modulated either by various transcription factors including C/EBPα, C/EBPβ, KLF5 and ZNF423 [40, 41] or by PPARγ-interacting proteins [40, 42]. Twist1, a basic helix-loop-helix (bHLH) transcription factor [12, 43], plays key regulatory roles in embryonic development, metabolic disease and cancer [44–46]. During mesoderm development, Twist1 is essential for lineage-specific differentiation [46, 47]. Involvement of Twist1 in cell proliferation and differentiation was also documented [48, 49]. For mesoderm-derived adipose tissue, Twist1 plays a regulatory role in adipose development [50, 51]. Through application of PPARγ agonist or antagonist, Ren et al. reported that the expression of Twist1 was responsive to PPARγ activity [21]. However, Ma et al. reported that Twist1 was up-regulated during adipocyte differentiation, and the expression of PPARγ was enhanced when Twist1 was knockdown at day 4 [52]. Pettersson et al. also reported that low expression of Twist1 in human white adipose tissue (WAT) was associated with obesity and insulin-resistance [19]. Thus, Twist1 may function as an anti-obesity factor during adipogenesis and this function is likely via the interaction between Twist1 and PPARγ. However, the exact mechanism of Twist1-regulated adipogenesis has not been profoundly elucidated.

In the present study, we identified Twist1 as a potent modulator of adipocyte differentiation. To confirm this conclusion, mesenchymal stem cell line C3H10T1/2 cell line [53] was used to recapitulate the lineage-specific differentiation of mesoderm development. As expected, Twist1 played a negative regulatory role in committed differentiation of C3H10T1/2, suggesting that Twist1 functions as a “brake” in commitment and subsequent differentiation of adipocytes. We further illustrated that Twist1 inhibits adipocyte differentiation via PPARγ, i.e., repressing either transcription or transcriptional activity of PPARγ. This is likely to be the underlying mechanism of Twist1-mediated suppression of adipogenesis. Furthermore, PPARγ and its partners also play important roles in adipogenesis [42, 54], including forming heterodimers with RXR [55, 56] and promoting adipogenesis [30]. Moreover, it is critical to clearly illustrate the interactions among Twist1, PPARγ and RXR under different biological conditions, although we speculated that Twist1 might be a competitor of RXR in binding PPARγ. In conclusion, we have delineated a regulatory relationship between Twist1 and PPARγ, which Twist1 binds to PPARγ protein or its promoter to repress either its transcriptional activity or expression, leading to attenuated adipocyte differentiation. In prospect, Twist1 might be a potential molecular therapy target and Twist1 agonist might be beneficial in controlling adipogenesis and harnessing obesity. However, we would also like to point out that the downregulation of Twist1 during adipocyte differentiation (days 0–6 for 3T3-L1 preadipocytes) and rebounded high-level expression of Twist1 in manure adipocytes (day 8 in mature 3T3-L1) implicate that Twist1 might play different functional roles in preadipocytes, during adipocyte differentiation and in mature adipocytes. Further studies are warranted to confirm this speculation.

### Supplementary information

Q-PCR primer sequences

Western blots

Reproducibility checklist

## Data Availability

The datasets generated and/or analyzed during the current study are available from the corresponding author on reasonable request.
