# Supplementary material for: Deciphering the interaction between Twist1 and PPARγ during adipocyte differentiation
Source: Cell Death Dis. 2023 Nov 23;14(11):764. doi: 10.1038/s41419-023-06283-0 (PMC10667345; doi:10.1038/s41419-023-06283-0)
Supplement: Supplementary file 1 — Q-PCR primer sequences [file 41419_2023_6283_MOESM1_ESM.docx]

**Table S1**. Q-PCR primer sequences

| Gene | Forward Primer | Reverse Primer |
| --- | --- | --- |
| Twist1 | CCCAGTCGCTGAACGAGG | TCCAGACCGAGAAGGCGTA |
| Pref1 | CCCGGCCATCTGCTTCAC | TCATGTCAATCTTCTCGGGGA |
| AdipoQ | TACTGCAACATTCCGGGACTC | TCCCCATCCCCATACACCTG |
| PPARγ | CTCACCGACGCACAGCA | GTGTGACTTCTCCTCAGCCC |
| aP 2 | AGATTTCCTTCAAACTGGGCG | AAACTCTTGTGGAAGTCACGC |
| C/EBPα | CTCTTCACTAACGGCTGGGG | TGGGTGGGGAAGCCTAAGT |
| C/EBPβ | GCCGCCTTATAAACCTCCCG | AGTCGGGCTCGTAGTAGAAGT |
| SREBP1 | CTCAGCAGCCCCTAGAACAAA | TGGTCCCTCCACTCACCAG |
| Itgbl1 | CTGGTTGGCATGGCGATAAAT | ACGTGTCCCCATGAATGTCTC |
| Pdgfrl) | GAGTGAACGCTATGGGCAGT | TGTCTGTCCGGGTTCAGGTA |
| Timp2 | TTCTTGTGCCGTGTTGATGC | ACAAAAGACGGGAGAGGCTG |
| Grem1 | GTCAAAGCGGGCACATTCAG | CTGTTTCCGGCTGGTGTTTG |
| Grem2 | CGTCATTGCAGGATGTTCTGG | TACTTGCGCTCGGTGACTAC |
| Scd1 | ACAACTACCACCACACCTTCC | AAACAGGAACTCAGAAGCCCAA |
| Scd2 | CACAACTACCACCACGCCTT | GGAACTGCAAGACCCCACAC |
| Klf15 | TTCAGTGTGACTTTGCTGTCATT | GGGAGCCTGGGACAGTAAAA |
| Fasn | GAGGGTGTGCCATTCTGTCA | CTAGAGGGCTTGCACCAACA |
| Lpin1 | GCCTGCTCGTGAATCCTCTT | AGCTGCCCCACGTAATTCAT |
| CA3 | TCTCTCTGGACCCTACCGAC | TCGCCTTTCTCCCGTCCTAT |
| Gck | CTCAAAAGCCATCCCCAAGC | GTTCACTTCTGGACCCCTCC |
| Dgat2 | ATTTGGCTACGTTGGCTGGT | CTTCAGGGTGACTGCGTTCT |
| Gpd1 | AATATAGTGGCCGTTGGGGC | CCCCATAGCAGGTCGTGATG |
| Acsl1 | GCCGCGACTCCTTAAATAGC | CCATGAGGGTGTTGGTTGGA |
