# Supplementary material for: Deciphering the interaction between Twist1 and PPARγ during adipocyte differentiation
Source: Cell Death Dis. 2023 Nov 23;14(11):764. doi: 10.1038/s41419-023-06283-0 (PMC10667345; doi:10.1038/s41419-023-06283-0)
Supplement: Supplementary file 2 — Western blots [file 41419_2023_6283_MOESM2_ESM.pdf]

Western blots

Figure 1

1A

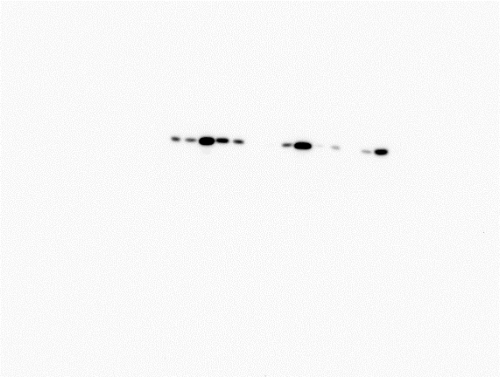

Twist1

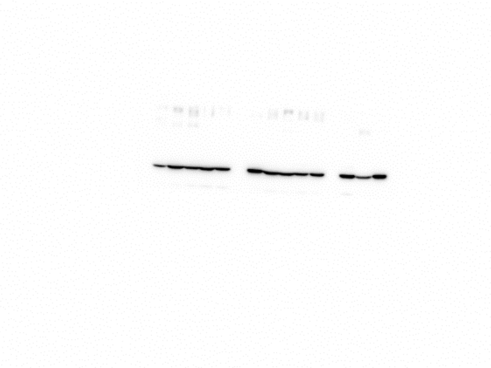

β-tubulin

1C

Adiponectin

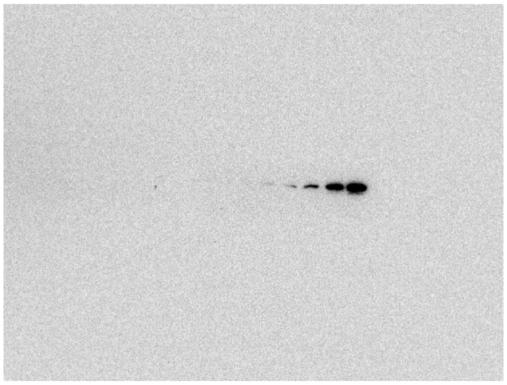

AP2

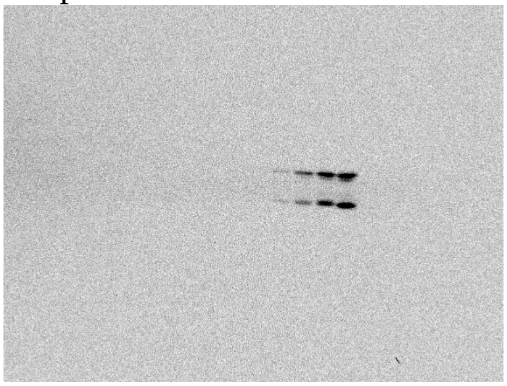

CEBPα

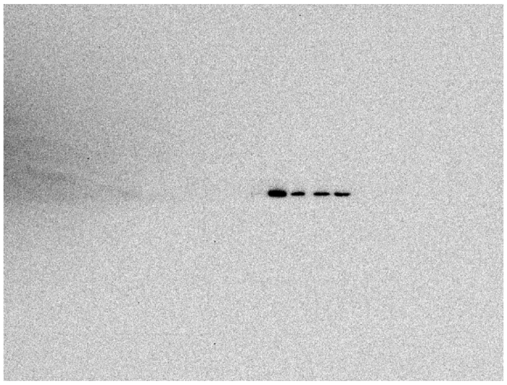

CEBPβ

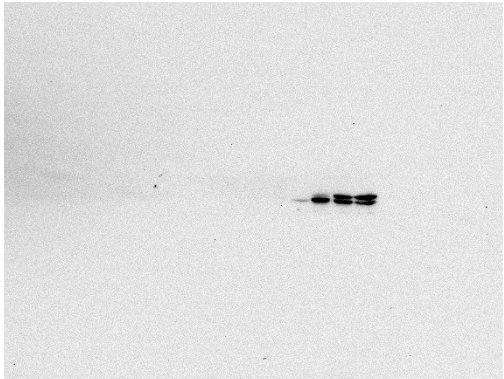
PPARγ

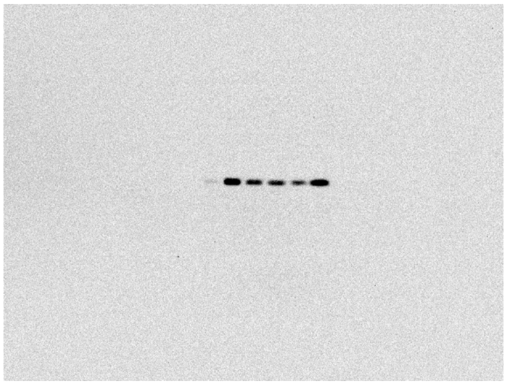
Twist1

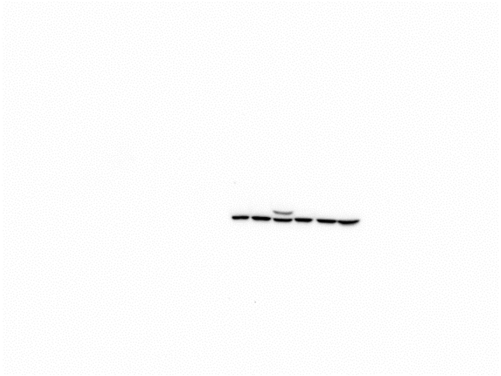
β-tubulin

Figure 2

2A

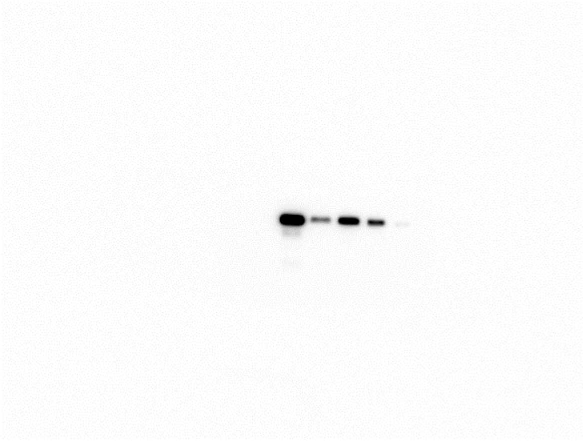

Twist1

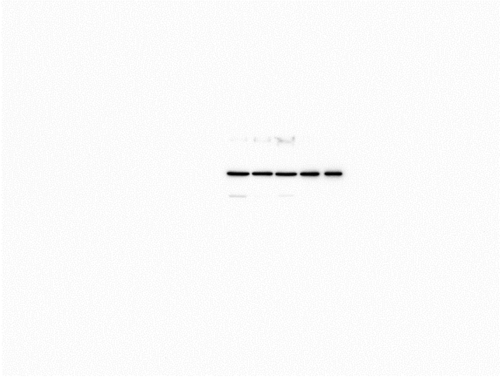

β-tubulin

2B

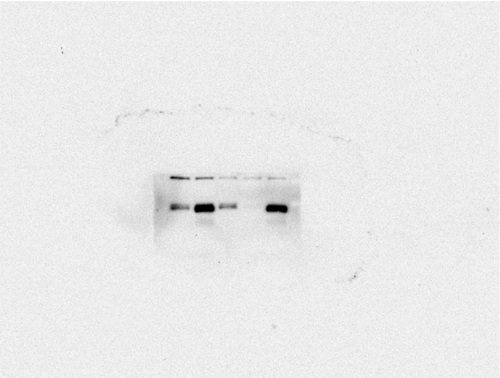

Twist1

β-tubulin

Figure 4

4B

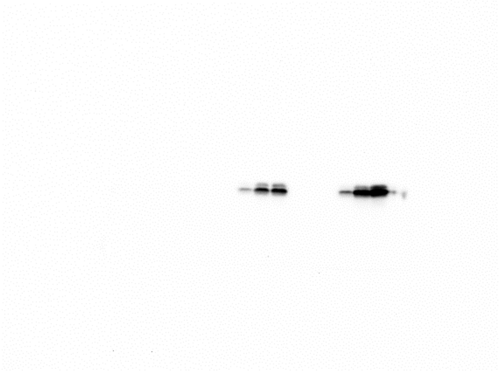

Adiponectin

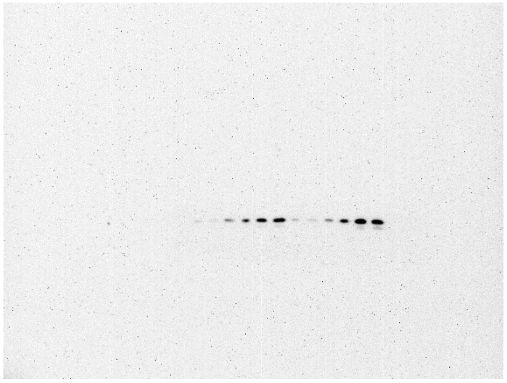

AP2

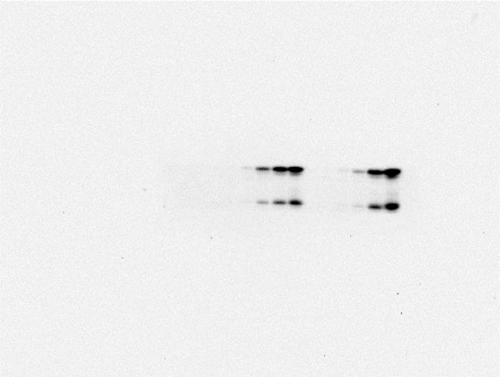

CEBPα

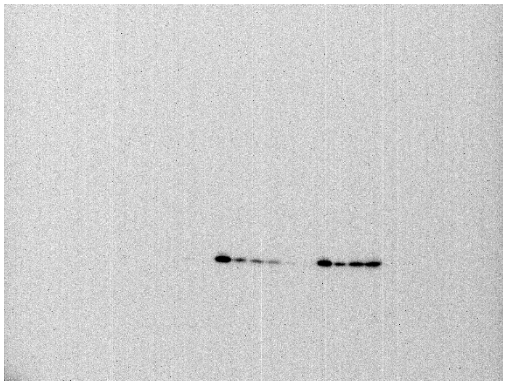

CEBPβ

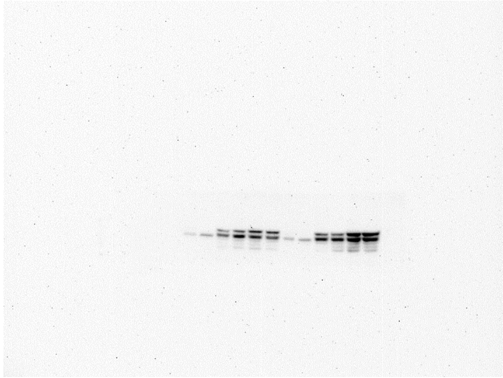

PPARγ

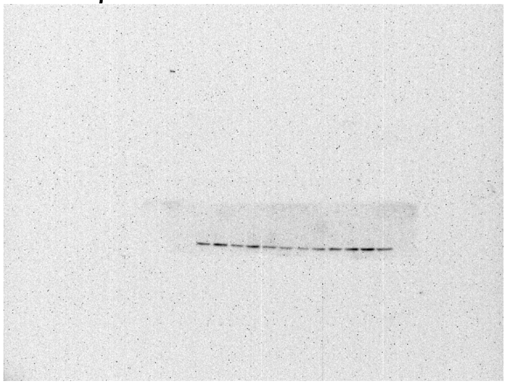

SREBP1

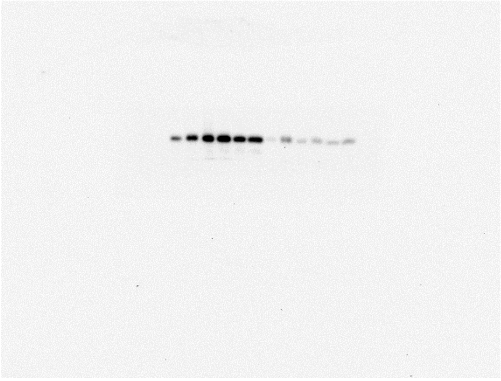

Twist1

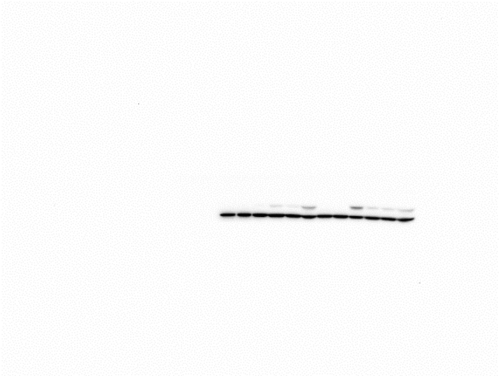

β-tubulin

4C

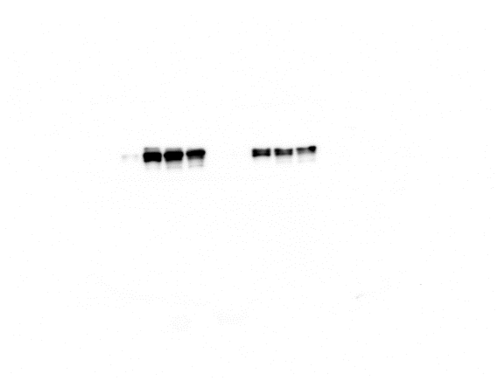

Adiponectin

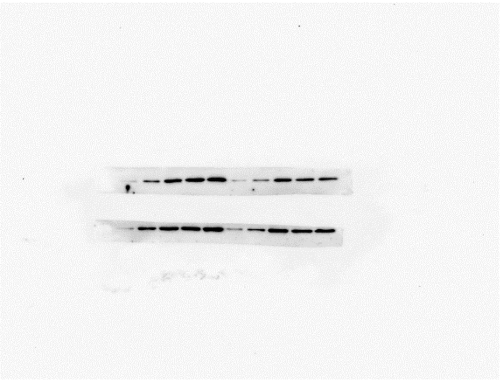

ap-8bit

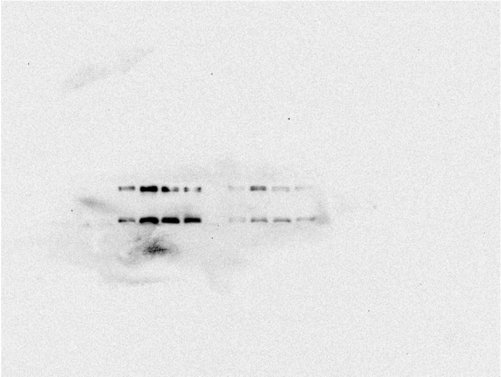

CEBPα

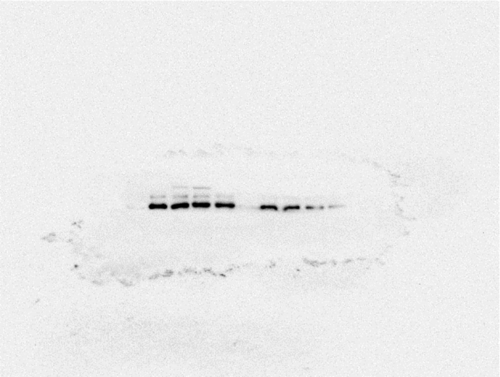

CEBPβ

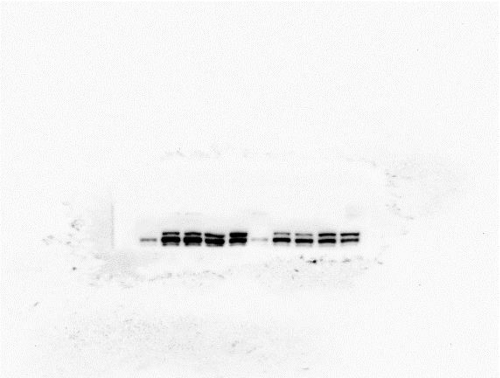
PPARγ

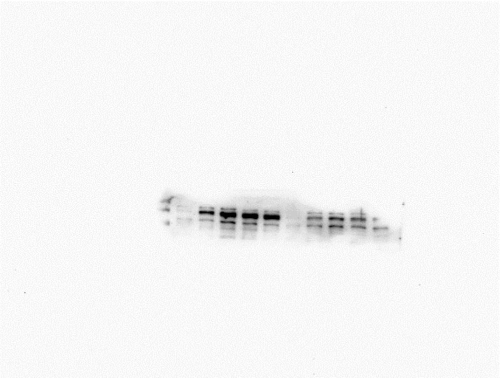
SREBP1

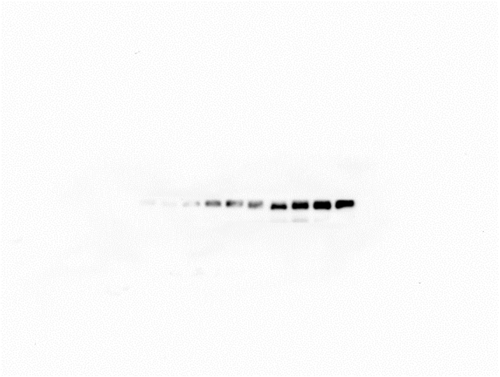
Twist1

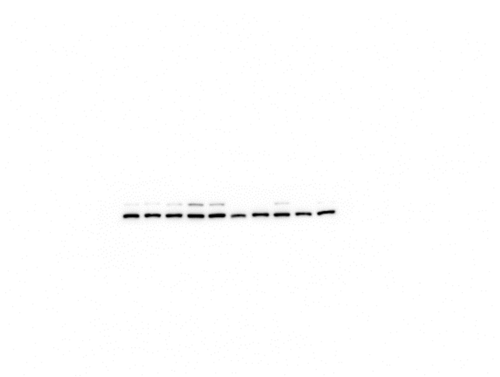
β-tubulin

Figure 6

6C

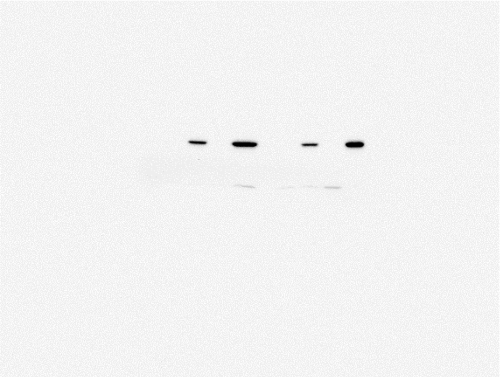

CA3

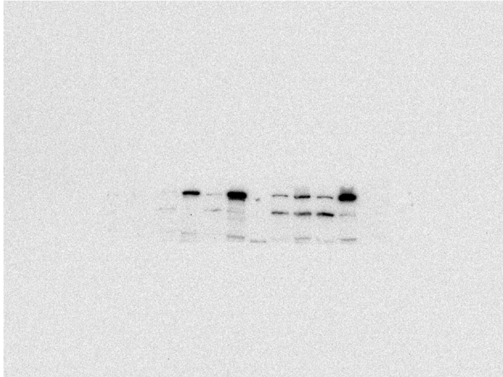

Fasn

Plin1

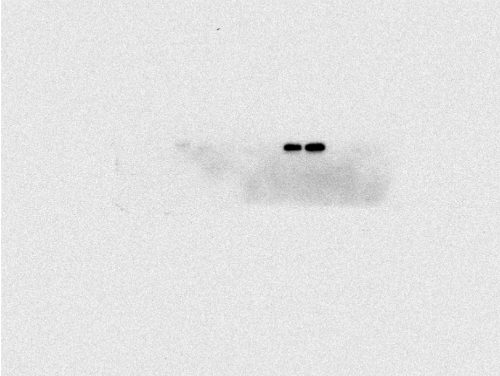

Twist1

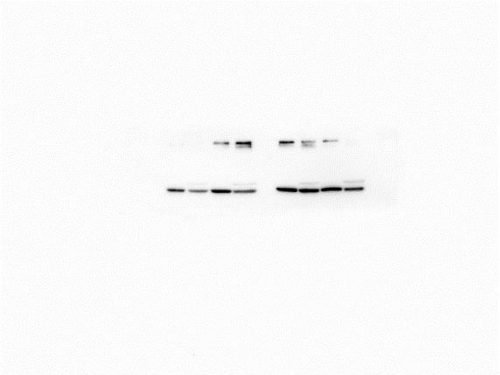

β-tubulin

6D

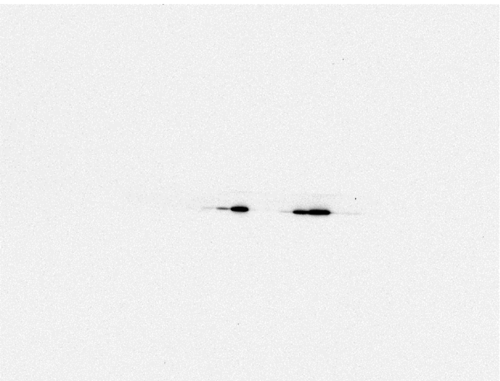

CA3

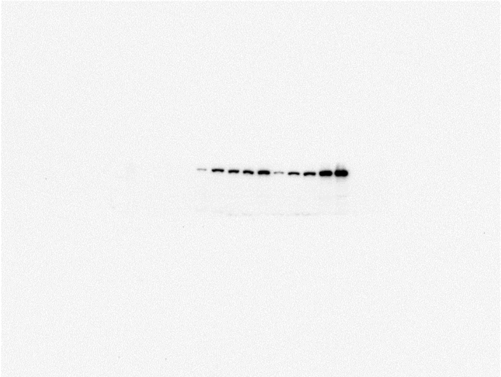

Fasn

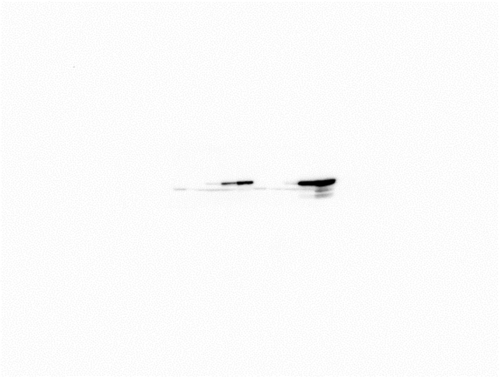

Plin1

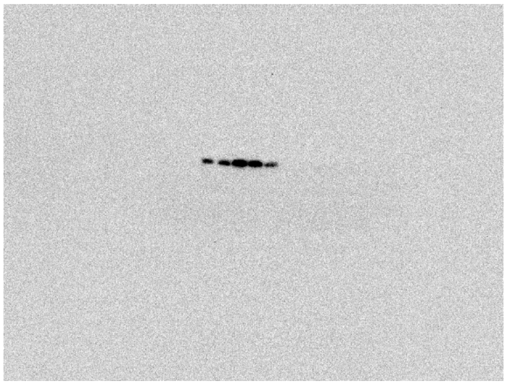

Twist1

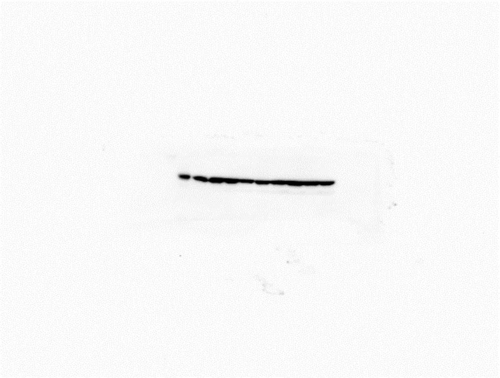

β-tubulin

Figure 7

7C

GAPDH

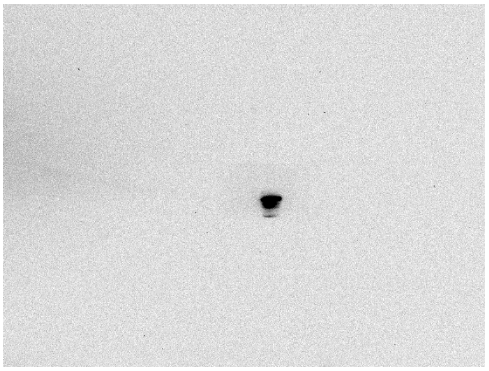

PPARγ

7E

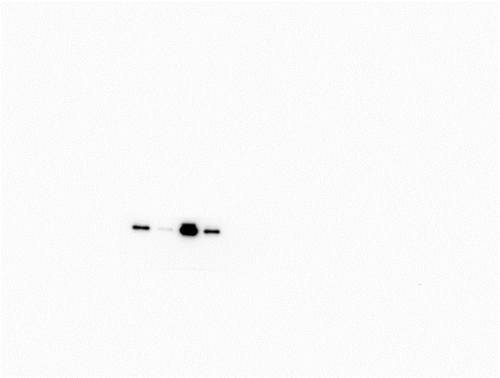

Adiponectin

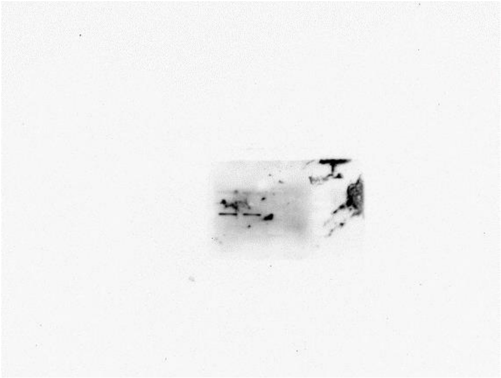

Lamin B1

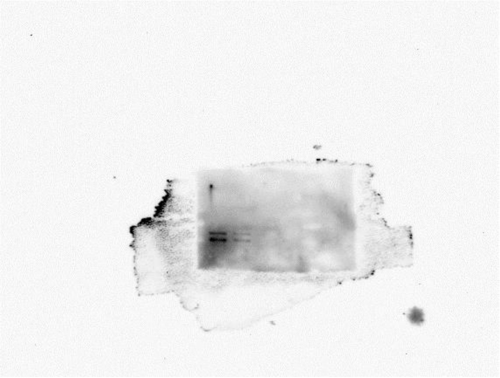

PPARγ

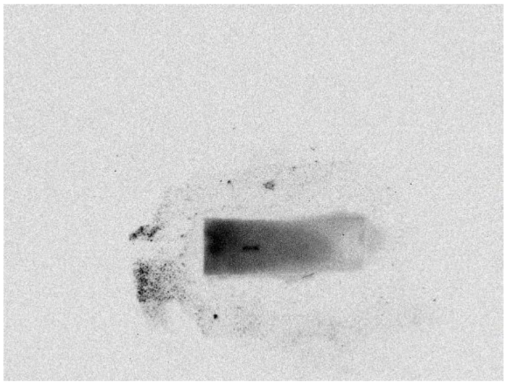

Twist1

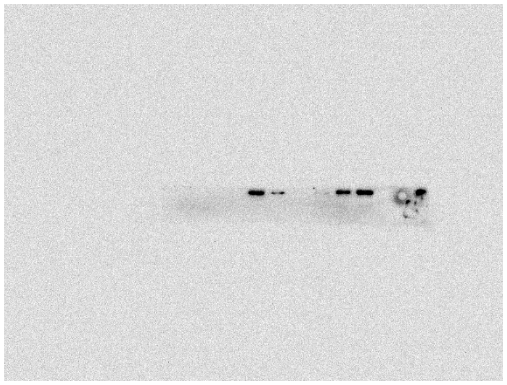

β-tubulin

Figure 8

8A

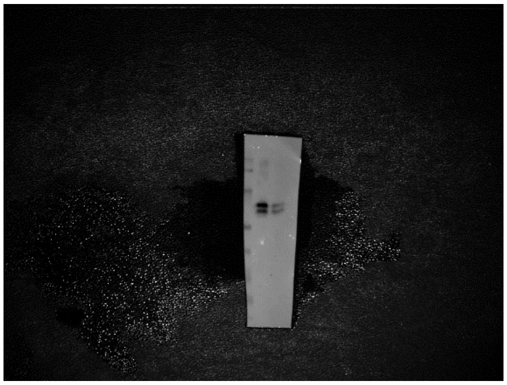

PPARγ

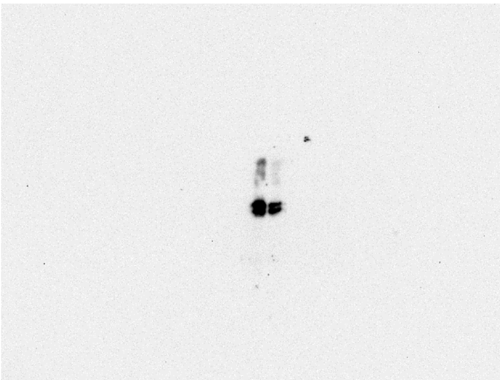

PPARγ-1

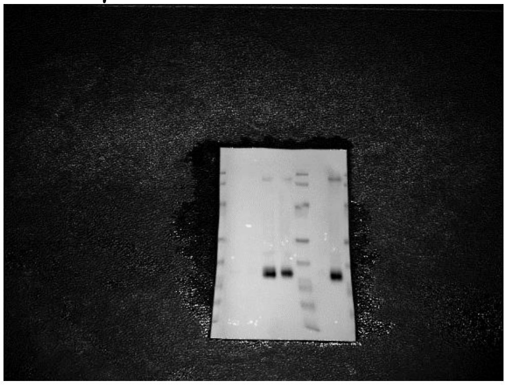

Twist1

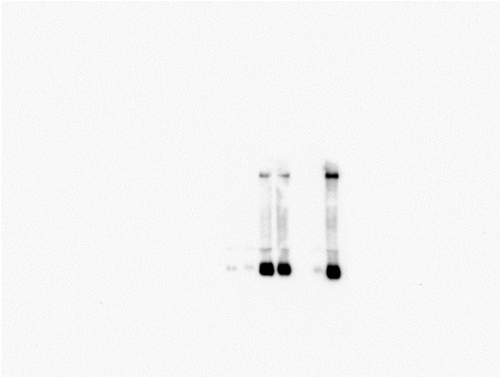

Twist1-1

8B

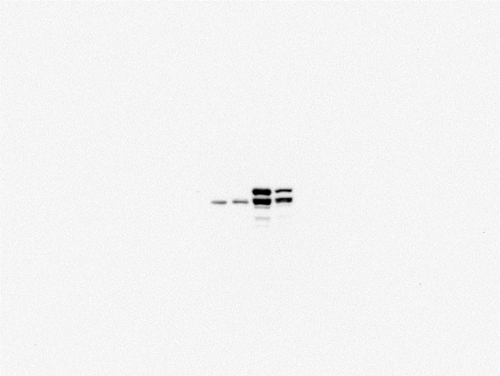

PPARγ

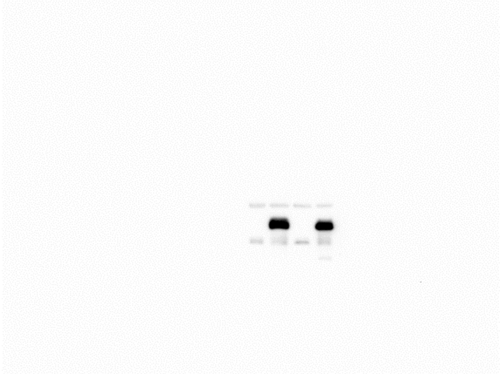

Twist1

β-tubulin

8C

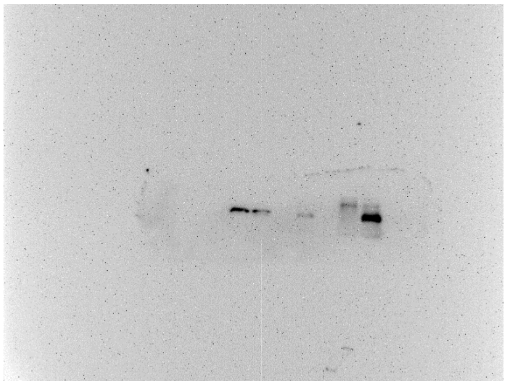

GFP- PPARγ

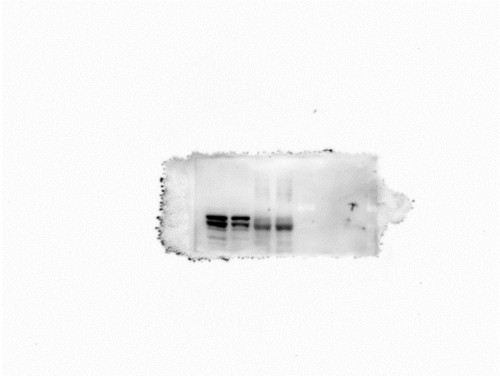

PPARγ

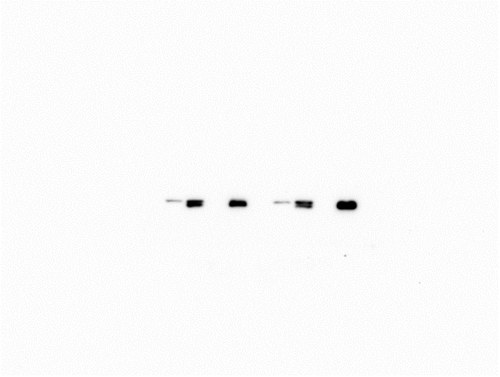

Twist1-1

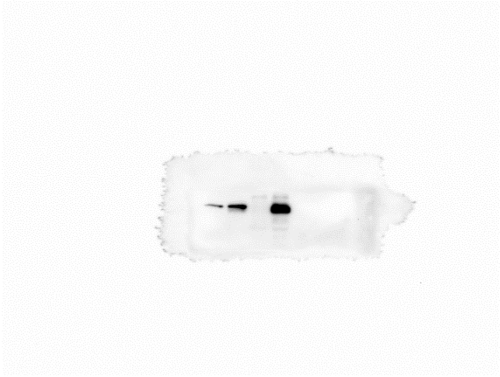

Twist1-2

8D

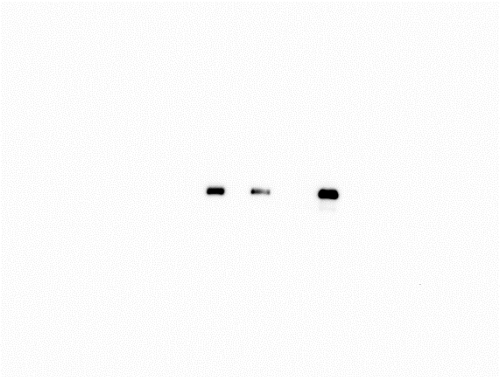

Flag-Twist1

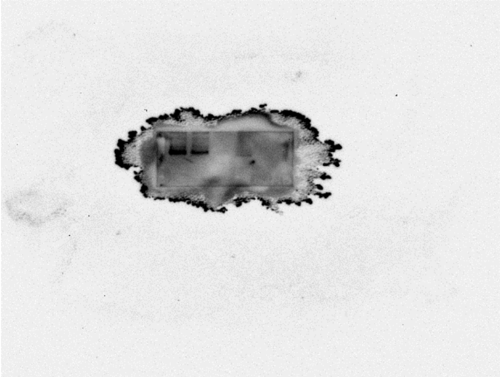

GFP-RXRα1-1

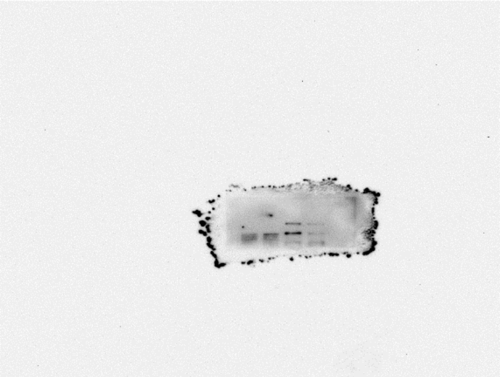

GFP-RXRα1-2

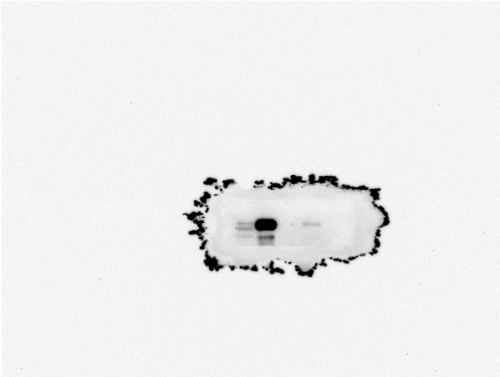

Twist1
